# Rapid Effects of Marine Reserves via Larval Dispersal

**DOI:** 10.1371/journal.pone.0004140

**Published:** 2009-01-08

**Authors:** Richard Cudney-Bueno, Miguel F. Lavín, Silvio G. Marinone, Peter T. Raimondi, William W. Shaw

**Affiliations:** 1 School of Natural Resources, University of Arizona, Tucson, Arizona, United States of America; 2 Institute of Marine Sciences, University of California Santa Cruz, Santa Cruz, California, United States of America; 3 Centro Intercultural de Estudios de Desiertos y Océanos (CEDO), Puerto Peñasco, Sonora, México; 4 Departamento de Oceanografía Física, Centro de Investigación Científica y de Educación Superior de Ensenada (CICESE). Carretera a Tijuana, Ensenada, Baja California, México; Monterey Bay Aquarium Research Institute, United States of America

## Abstract

Marine reserves have been advocated worldwide as conservation and fishery management tools. It is argued that they can protect ecosystems and also benefit fisheries via density-dependent spillover of adults and enhanced larval dispersal into fishing areas. However, while evidence has shown that marine reserves can meet conservation targets, their effects on fisheries are less understood. In particular, the basic question of if and over what temporal and spatial scales reserves can benefit fished populations via larval dispersal remains unanswered. We tested predictions of a larval transport model for a marine reserve network in the Gulf of California, Mexico, via field oceanography and repeated density counts of recently settled juvenile commercial mollusks before and after reserve establishment. We show that local retention of larvae within a reserve network can take place with enhanced, but spatially-explicit, recruitment to local fisheries. Enhancement occurred rapidly (2 yrs), with up to a three-fold increase in density of juveniles found in fished areas at the downstream edge of the reserve network, but other fishing areas within the network were unaffected. These findings were consistent with our model predictions. Our findings underscore the potential benefits of protecting larval sources and show that enhancement in recruitment can be manifested rapidly. However, benefits can be markedly variable within a local seascape. Hence, effects of marine reserve networks, positive or negative, may be overlooked when only focusing on overall responses and not considering finer spatially-explicit responses within a reserve network and its adjacent fishing grounds. Our results therefore call for future research on marine reserves that addresses this variability in order to help frame appropriate scenarios for the spatial management scales of interest.

## Introduction

As a response to declining fish stocks and threats to marine ecosystems, marine reserves (areas closed to fishing) have been widely advocated as conservation tools and means to achieving more sustainable use of marine resources [1]–[3]. The rationale behind their use lies in the dual opportunity they could offer to protect ecosystems and ecological processes while also enhancing fisheries via density-dependent spillover and larval dispersal of target species into fishing areas [1], [3]–[5]. However, while evidence has shown that marine reserves can meet conservation targets [6]–[8], the role they may have on fisheries is less understood. Previous studies have focused on benefits to adjacent fisheries via density-dependent spillover of adult fish from reserves [6], [9] or have been based primarily on larval transport models [3], [10]–[12], lacking validation through field monitoring and oceanographic data.

Models that inform effects of marine reserves via enhanced larval export rely on (a) assumptions about recruitment limitations in unprotected populations, and (b) connectivity between reserves and non-reserve sites [10]–[12]. If both assumptions are met, marine reserves could replenish adjacent, fished populations. However, effects could be localized or widespread depending on dispersal, which is in turn related to the complex interactions among local current patterns and larval duration and behavior [13]–[16]. Hence, actual effects are difficult to measure and understand. Furthermore, without explicit model predictions of patterns of enhanced recruitment, assumptions of reserve effects can neither be supported nor falsified by empirical results. These have been fundamental problems in investigations of marine reserves [17], and the basic question of if and over what temporal and spatial scales reserves can benefit fished populations via larval dispersal remains unanswered.

**Figure 1:**
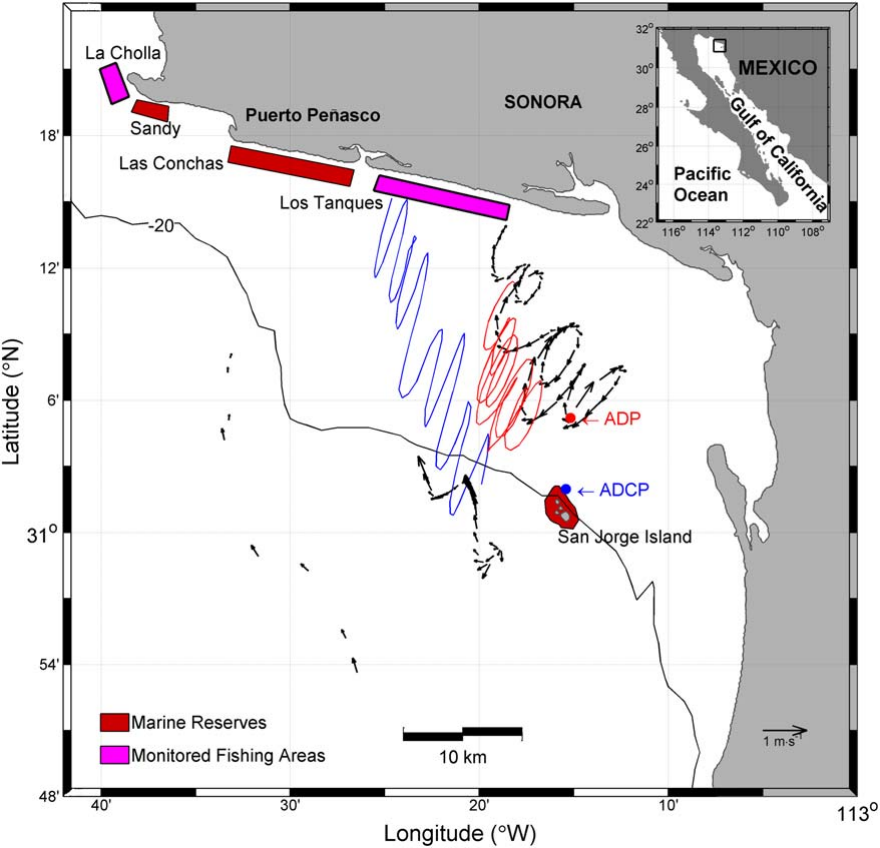
Location of reserve network, monitored fishing areas, and observations of regional currents. The three diagrams in the center represent: track and velocity of a surface drifter (in black), progressive vector diagram (PVD) from velocity measured 15 m above the bottom at ADCP (Acoustic Doppler Current Profiler) site (in blue, bottom at 25 m), and the PVD from velocity measured 15 m above the bottom at ADP (Acoustic Doppler Profiler) site (in red, bottom at 18 m). Both PVDs have been shifted west for clarity. These three diagrams are made with hourly-mean data for the period 19:00:00 (UT) July 12 to 00:10:00 July 16 2006. The black arrows close to the 20 m isobath represent interpolated half-hourly data from a drifter drogued at 15 m, from 03:00 to 22:30, July 7 2006. The most offshore arrows are 6-hourly drifter data from the same drifter, from 18:00 June 24 to 12:00 June 25 2006 (see Fig. S1 and S2 for additional drifter trajectories and PVDs).

As a means to test the effects of reserves on adjacent fisheries via larval dispersal, we coupled predictions from a larval transport model with *in situ* field oceanography and monitoring of densities of individuals recruited since the establishment of a reserve network in Northwest Mexico. The Puerto Peñasco reserve network was established in summer 2002 primarily as a means to protect declining stocks of two commercial species of mollusks: rock scallop (*Spondylus calcifer*) and black murex snail (*Hexaplex nigritus*). The network includes an offshore reserve (San Jorge Island), and two coastal reserves (Las Conchas and Sandy) (Fig. 1). It covers approximately 18 km of coastline composed primarily of extended beach-rock (coquina) and granite reefs separated by beds of mussels and rhodoliths and shell/sandy patches.

## Results

To assess the effects of the reserve network, we generated a larval export model and tested its predictions through observations of currents and bi-annual density counts of juvenile rock scallops and murex snails within reserves and fishing grounds prior and after reserve establishment (summer 2002-summer 2004). We developed a three-dimensional baroclinic numerical model that was based on the circulation pattern for the summer (the spawning season for both species), which is cyclonic overall [18], with northwestward flow in the area where the reserve network is located. We used the model to assess if the network could receive larvae from southern sources and to predict patterns of larval recruitment within the network. The model tracked passive particles for up to four weeks (exceeding the range of larval duration for both species) after being released (a) in the rocky reef south of the reserve network (∼150 km south of San Jorge Island), (b) in San Jorge Island, the southern boundary of the network, and (c) every km from the Island to the northwestern portion of the network.

**Figure 2:**
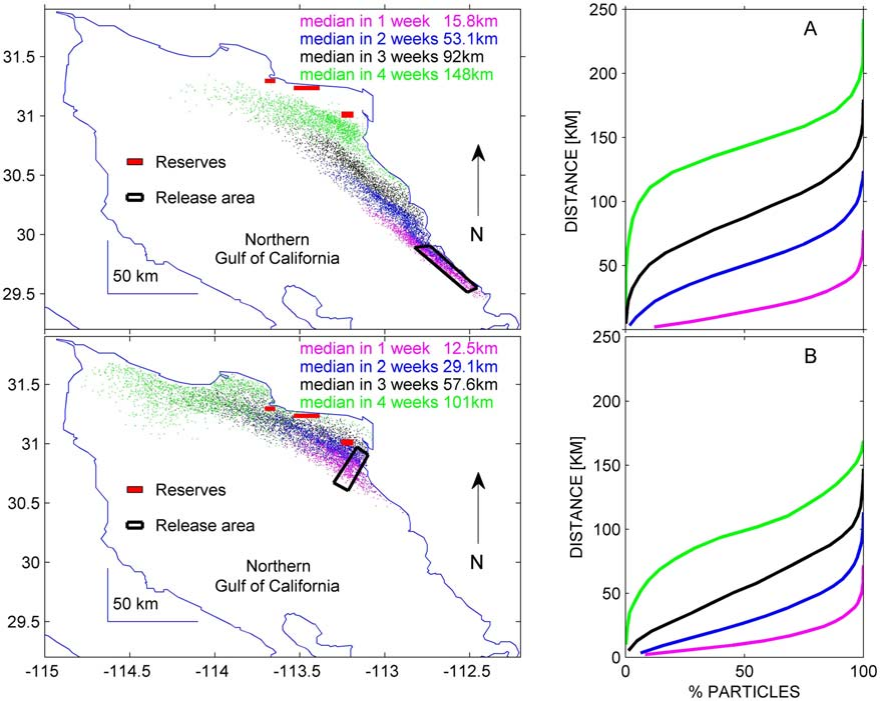
Final position of particles 1–4 weeks after having been released in (a) the nearest rocky reef located south of the marine reserve network, and (b) the network's southern boundary (San Jorge Island). Panels on right show cumulative percentages of particles as a function of distance 1, 2, 3 and 4 weeks after release.

In case (a) released particles showed a median south-northwest travel distance of 148 km in four weeks (Fig. 2A). Larvae of both species, however, are competent to settle in less time [19], [20]. It is therefore highly unlikely that there could have been any substantial direct influence from southern reefs, particularly on coastal reserves which are 180–200 km north of these reefs. Furthermore, our model predictions are likely an overestimation of true dispersal distances, as larvae dispersal can be more constrained once behavior and habitat are accounted for in model predictions [15], [21]. Influence from western sources (Baja California peninsula) is highly unlikely, as previous studies indicate a clear cyclonic movement of the water during summer [22], when both of these species reproduce [23], [24]. On the eastern side of the Gulf of California, the water mass has a northbound movement whereas on the western side water moves towards the south and does not reach the eastern coastline [22].

**Figure 3:**
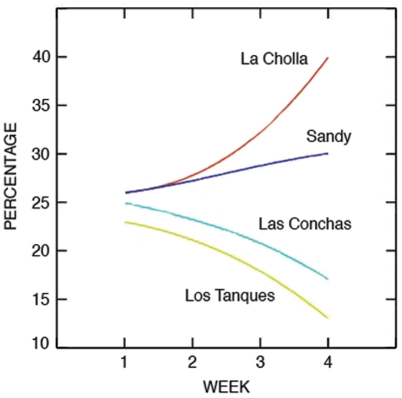
Modeled larval settlement (as relative percentages) within coastal reserves (Las Conchas, Sandy) and fishing areas (Los Tanques, La Cholla) as a function of the day larvae are competent to settle. Model larvae were released every kilometer in the region of interest, from San Jorge Island to La Cholla. Earlier results suggested that there was no source of larvae to the south of the network. No sources were used to the north because models showed that larvae released to the north of the reserve network would be transported away from the network. The model assumed that larvae settled on the day of competency. If that assumption is relaxed, the difference in settlement between northern (Sandy/La Cholla) and southern (Las Conchas/Los Tanques) sites increases.

Particles released in the area surrounding San Jorge Island (case b), the southern portion of the reserve network, showed a marked flow toward the coast and northwestern reserve sites (Fig. 2B). Direct evidence of this flow pattern is provided by the tracks of surface drifters released near the Island and progressive vector diagrams (PVDs) from concurrent acoustic current profilers (ADCPs, ADPs) (Fig. 1; also, see Fig. S1 and S2). Drifter tracks show the tidal ellipses plus a residual flow toward the coast (tidal ellipses refer to the trajectory that drifters followed with the ebb and flow of the tide; while residual flow refers to the net displacement of drifters over one or more tidal cycles, in this case, progressively moving north toward the coast). When modeling larval settlement as a function of distance from the Island to coastal reserves and monitored fishing areas (case c), for any period from 1–4 weeks the model predicted more settlement at northernmost sites (Sandy/La Cholla) (Fig. 3). Following this same modeling exercise, more settlement in southern reserve and fishing areas (Las Conchas/Los Tanques) compared to northern ones would only be evident if larvae were competent to settle no more than two days after release. However, larvae of both species are planctonic and competent to settle in >1 week [19], [20].

**Figure 4:**
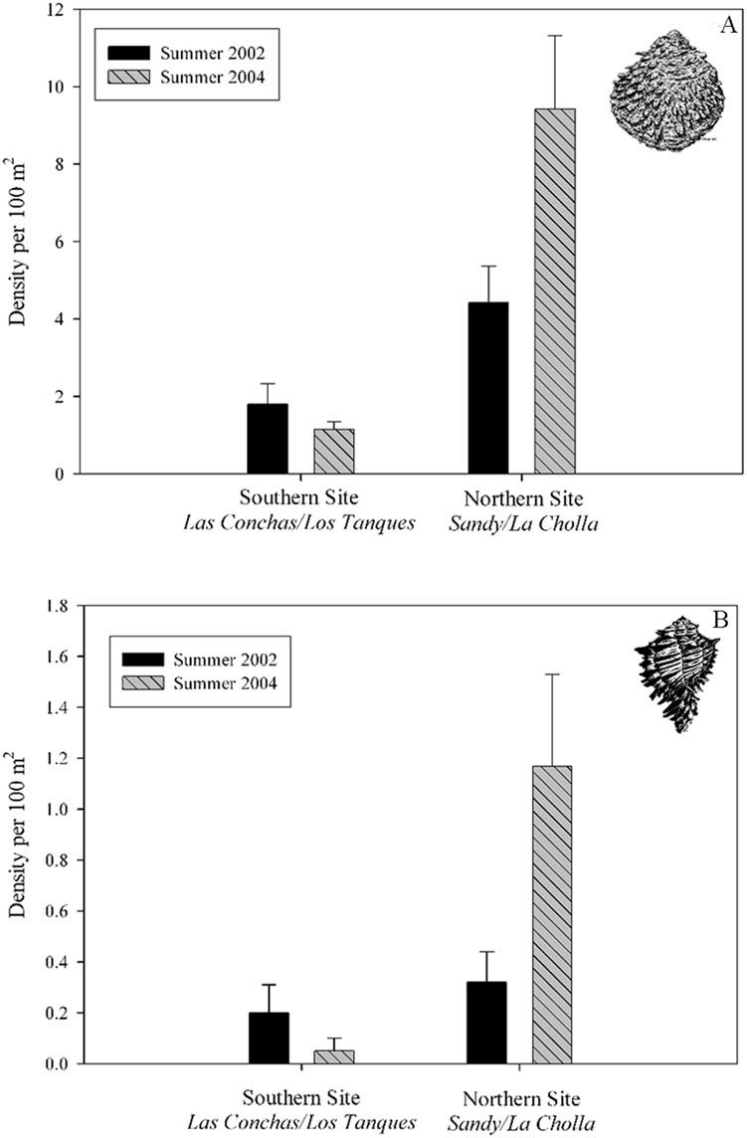
Differences in densities (S.E. bars included) of juvenile rock scallops (a) and black murex snails (b) in southern and northern sites before (summer 2002) and after (summer 2004) reserve establishment.

Observed spatial patterns of recruitment of juveniles of both species (individuals born and recruited since reserve establishment) were consistent with predictions of our larval transport model. Only two years after establishment of the reserves, both species showed evidence of changes in density as a function of time, protection from fishing (reserve effects) and site effects as a whole (repeated measures three-way MANOVA, time X protection X site; rock scallop: Pillai's Trace F_4, 41_ = 2.53, P = 0.05; black murex: Pillai's Trace F_4, 41_ = 3.02, P = 0.02). Density of juvenile rock scallop had increased by up to 40.7% within coastal reserves and by 20.6% in fished areas (repeated measures two-way MANOVA, time X protection from fishing, Pillai's Trace F_4, 41_ = 2.67, P<0.05). Changes were also evident for black murex, with more than a three-fold increase in density of juveniles within fished areas (repeated measures two-way MANOVA, time X protection from fishing, Pillai's Trace F_4, 41_ = 3.28, P<0.05). The pattern of increase in juveniles, however, was variable in space, evident only for the northwestern portion of the network (Fig. 4), as predicted by the larval transport model for any period between one and four weeks. Density of both species increased markedly in the reserve and fished northwestern sites (Sandy/La Cholla) and remained relatively constant in southeastern sites (Las Conchas/Los Tanques) (repeated measures two-way MANOVA, time X site; rock scallop: Pillai's Trace F_4, 41_ = 7.09, P<0.001; black murex: Pillai's Trace F_4, 41_ = 2.95, P<0.05) (Table S1 and S2).

## Discussion

Observed increase in recruitment was spatially-constricted to the northern portion of the reserve network and consistent with predictions of our larval transport model and field oceanographic observations. This recruitment pattern reflects effects of larval dispersal within the reserve network rather than other effects such as density-dependent adult spillover, a good year of high overall regional recruitment, or wider oceanographic processes. The restricted adult movement of both species (sessile in the case of rock scallop) constrains our inference to larval dispersal rather than density-dependent adult spillover. Furthermore, high overall recruitment and regional oceanographic processes would likely have resulted in changes throughout the study area, not only in the northern portion of the reserve network.

Surveys on San Jorge Island, as well as our modeling and current measurements (Figs. 1 and 2B) suggest that the Island could be acting as a key component of the network, providing a source for larval export to adjacent coastal reserves and fishing areas. Overall density of juvenile rock scallops on the Island actually decreased since reserve establishment (repeated measures 1-way ANOVA; F_4, 45_ = 4.46, P<0.01) and those of black murex remained relatively constant (repeated measures 1-way ANOVA; F_4, 45_ = 0.615, P = 0.65). However, although density of juveniles did not increase, even the lowest average numbers in five monitoring seasons were 80% higher than those of coastal reserves. Overall densities (adults and juveniles) were also six times higher than those of all coastal reserves and fishing sites combined, reaching up to 1.6/m^2^ and exceeding any others reported for the Gulf of California [23], [25]. Given these high densities, a decrease in juveniles near the Island could be related to density-dependent processes.

Our findings provide needed insights for theory and empirical understanding of effects of marine reserves. First, we show evidence of rapid effects of reserve networks on adjacent fisheries via larval dispersal. Second, we also show that local retention of larvae within a network can take place with enhanced but spatially variable recruitment to local fisheries. Hence, effects should not be expected across an entire reserve network. Rather, they can be markedly variable within a local seascape.

These results have important implications for management. Reserves reduce the total area available for fishing, likely causing an initial economic cost to fishers. Therefore, in situations where there is local support for reserve establishment, evidence of rapid positive reserve effects, as we have here shown, could play a crucial role in reinforcing cooperation among fishers for further compliance [26]. Evidence of larval retention and enhanced recruitment to local fisheries also underscores the benefits of protecting reproductive larval sources and reconciles local management with social needs. Reserve networks with strong support from fishing communities are best designed if they enhance or maintain recruitment within the area of influence of these communities, not benefiting others at the expense of local management initiatives and, ultimately, initial costly decisions. In some situations, however, this may not be possible, as oceanographic processes could result in larval export outside the area of influence of the community or communities supporting the reserve network. Designs of reserve networks that cover broader spatial scales may be needed in these situations.

Finally, effects of marine reserves, positive or negative, may be overlooked when only focusing on overall responses and not considering finer spatially-explicit responses within a reserve network and its adjacent fishing grounds. Our results therefore call for future research on marine reserves that addresses this variability in order to help frame appropriate scenarios for the spatial management scales of interest. Not doing so could lead to false expectations among stakeholders.

## Materials and Methods

### Particle tracking from a three dimensional oceanographic numerical model

We released 2000 passive particles in two areas (between 0–60 m deep): San Jorge Island, and the nearest substantial rocky reef south of the marine reserve network. Particles were tracked for four weeks and the temporal scales resolved by the model (due to forcing) are tidal and seasonal. The model is described in detail for the Gulf of California by Marinone [27] and Mateos et al. [28]. Briefly, the model domain has a mesh size of 2.5′×2.5′ (∼3.9×4.6 km) in the horizontal and 12 layers in the vertical with nominal lower levels at 10, 20, 30, 60, 100, 150, 200, 250, 350, 600, 1000 and 4000 m. Model equations are solved semi-implicitly with fully prognostic temperature and salinity fields. The model is forced with tides, climatological winds, climatological hydrography at the mouth of the Gulf of California, and climatological heat and fresh water fluxes at the air-sea interface. As shown by Marinone [27], the model adequately reproduces the main seasonal signals of surface temperature, heat balance, tidal elevation and surface circulation in the northern Gulf of California and also the tidal currents as shown by Marinone and Lavín [29].

### Drifter tracks and current profiles

We used two bottom-mounted acoustic current profilers moored at the sites marked ADCP and ADP in Fig. 1 and six PacificGyre (www.pacificgyre.com/Lagrangian.aspx) Microstar surface drifters which drogues were centered at 1 m below the sea surface. These drifters provided GPS positions every 10 minutes. One PacificGyre ARGOS SVP (Surface Velocity Program) drifter was used to observe the current field offshore (west) of San Jorge Island; it was drogued with a 4.8 m- tall Holey Sock centered at 15 m depth.

Site ADCP (Fig. 1), equipped with a bottom-mounted 300 KHz Acoustic Doppler Current Profiler (by RDInstruments), was set just north of San Jorge Island where the mean bottom depth was 25 m. Site ADP, which contained a bottom-mounted 500 KHz Acoustic Doppler Profiler (by SonTek), was 8 km further north with the bottom at ∼18 m. Both current profilers measured the mean velocity of every meter of the water column, every three minutes, for the periods June 2-July 4 and July 6-August 18 2006. For the purpose of this article, the best way to present the profiler current data are the Progressive Vector Diagrams (PVD), which are constructed by calculating the vector displacement that a water parcel would experience at the mooring position during each sampling interval, and drawing them sequentially, the tail of each vector on the head of the previous one. Note that they are not true tracks, but they can be plotted over maps to aid in the interpretation. The PVDs in Fig. S1 correspond to the ADCP data at 3.6, 9.6, 15.6 and 20.6 m above the bottom (cells 1, 7, 13 and 18) for the period July 6 to July 30 2006; they show the tidal ellipses plus a residual to the NW (∼2 cm/s). This pattern was consistent throughout the observation periods. In Fig. 1 we plotted the PVDs at 15 m above the bottom for both the ADCP and the ADP, from 19:00 UT on July 12 to 00:10 UT on July 16 2006. The surface drifters were deployed in groups of 4–6 units; several deployments were made between July 12 and July 23 2006, either over the ADP or ADCP sites or off the northern end of San Jorge Island. The tracks of 5 drifters (and their velocities) for the period July 12 (19:00 UT) to July 16 (00:10 UT) are shown in Fig. S2; in addition to the (true) tidal ellipses, the tracks also show a residual flow to the NW. In Fig. 1, the track and velocity of one of the drifters is plotted together with the PVDs from the two current profilers, for the period covered by the drifter track. Fig. 1 also shows the tracks and velocities obtained outside San Jorge Island with the SVP drifter. Two tracks are shown, the most offshore comprises 6-hourly data from 18:00 (UT) June 24 to 12:00 (UT) June 25 2006, and the second interpolates half-hourly data from 02:57 (UT) to 22:23 (UT), July 7 2006.

### Estimation of population parameters

We estimated changes in density of rock scallop (*Spondylus calcifer*) and black murex snail (*Hexaplex nigritus*) in reserve and fishing sites for two consecutive years beginning in May 2002, one month preceding reserve establishment. The region monitored encompassed the reefs of San Jorge Island and those found near the fishing town of Puerto Peñasco (within 3 km from highest tide line) in the eastern part of the northern Gulf of California, Mexico. This region extends from 31,22,18.1 N; 113,39,09.4 W to 31,15,03.8 N; 113,20,48.1 W.

We subdivided the region into 5 sampling areas: a) two coastal reserves (replicates), Las Conchas and Sandy; b) two coastal fishing areas (“controls”), Los Tanques and La Cholla; c) one offshore island reserve, San Jorge Island (Fig. 1). We paired coastal reserves with appropriate coastal fishing areas (Sandy with La Cholla; Las Conchas with Los Tanques). We refer to each of these pairs as “sites”. Given the lack of adequate comparison areas for San Jorge Island, we analyzed the response of this off-shore reserve independently. Assessments are based on bi-annual density counts in 58 100 m^2^ permanent plots before and after reserve establishment (repeated measures, five monitoring seasons).

To reduce heterogeneity associated with depth, we restricted all sampling to depths ranging from 40–65 ft. This also reduced health risks associated with diving and facilitated overall monitoring as we were able to remain underwater for longer periods of time. In all cases except San Jorge Island, this depth as well as the established constrained distance from the tide line covers the entire extension of the reefs. We restricted sampling in San Jorge Island to the reefs found on the eastern part of the island, as these are shallower and more similar to those found on the mainland coast.

### Plot design and sample unit selection

We selected plots from within these five areas through simple random sampling. In the event that a specific plot selected happened to fall where at least 50% of sand was present, that plot was replaced by another one by swimming underwater in a straight line along the reef until reaching sufficient (>50%) rocky substrate. Once selected, all plots were permanently marked underwater.

Plots were 10×10 m subdivided into 16 quadrats of 2.5×2.5 m for ease of observation. Testing other sampling methods such as the use of 5×50 m or 5×30 m transects, distance sampling or others typically used for sessile organisms did not prove adequate for this region given the highly variable visibility of the region, the strong currents, and the overall patchiness of the reefs (i.e. patches of reef typically separated by patches of sand). We counted all individuals visible within each 2.5×2.5 m quadrat (subplot). For rock scallop, we estimated size of each individual to fall within one of three categories: small juveniles (up to 5 cm of height), medium-sized juveniles and young adults (>5 and <10 cm of height), and large adults (>10 cm of height). For black murex, sizes fell into two categories: juveniles and reproductively mature adults.

To reduce variation in detectability, the same person counted organisms on each sampling occasion and in the same designated plots while another diver assisted setting and maintaining the plot lines in place. To support this work, ten commercial divers with extensive experience searching for benthic mollusks (>5 years) were trained to participate in the monitoring process. We calculated variations in the detection of monitored species (s≤3 individuals/plot) and incorporated this variation to calculate statistical power of our sampling design (see below).

### Sampling frequency, sample sizes, and allocation of samples

We established a total of 58 sampling plots: San Jorge = 10, Las Conchas = 10, Los Tanques = 10, Sandy = 10, La Cholla = 18. Power analyses from baseline data on density of rock scallops found on these 58 plots gave us a high probability of detecting at least a 10% increase in their density (Power>95% for each reserve and fishing zone, α = 0.05 and s = 3 individuals/plot). Given that rock scallops are harder to detect than black murex (when closed they resemble rocks), we assume an even higher statistical power for detection of changes in population densities of black murex. We monitored each plot twice every year (Spring and Summer) for two consecutive years (Summer 2002, Spring 03, Summer 03, Spring 04, Summer 04). These months provide some of the best visibility underwater and are also usually devoid of algae beds covering the rocky reefs, which reduce detectability of species monitored.

### Statistical analysis

This is a longitudinal study with a repeated measures research design and various levels of analysis. We first generated profile plots of baseline data and applied square root transformations to improve homogeneity of variance. We then addressed “between subject” and “within subject” variability of baseline data graphically, determined the coefficient of variation, and tested for independence of plots and sampling sites in order to avoid pseudo-replication. We used multivariate analyses of variance (MANOVA) and relied on Multivariate Pillai's Trace P values to help assess time, protection from fishing, and treatment effects independently as well as combined factors. Univariate estimates were also obtained and analyzed to further understand observed patterns (see Table S1 and S2).

## Supporting Information

Table S1 Univariate and multivariate tests for the analysis of temporal changes in density of juvenile rock scallops found within monitored reserve and fishing areas.
(0.05 MB DOC)

Table S2 Univariate and multivariate tests for the analysis of temporal changes in density of juvenile black murex found within monitored reserve and fishing areas.
(0.05 MB DOC)

Figure S1 Progressive vector diagrams (PVD) calculated from the ADCP velocity data at 3.6, 9.6, 15.6 and 20.6 m above the bottom (cells 1, 7, 13 and 18) for the period July 6 to August 18 2006. For clarity, successive diagrams are shifted to the left by 5 km.
(0.13 MB TIF)

Figure S2 Tracks and velocities of the Microstar surface drifters for the period 19:00 (UT) July 12 to 00:10 July 16 2006. Four of the five drifters shown were redeployed during the period; the exception is the green trace, which is used in Fig. 1 of the article.
(0.02 MB TIF)
