# Supplementary material for: Rapid Effects of Marine Reserves via Larval Dispersal
Source: PLoS One. 2009 Jan 8;4(1):e4140. doi: 10.1371/journal.pone.0004140 (PMC2612740; doi:10.1371/journal.pone.0004140)
Supplement: Table S1 — Univariate and multivariate tests for the analysis of temporal changes in density of juvenile rock scallops found within monitored reserve and fishing areas. (0.05 MB DOC) [file pone.0004140.s001.doc]

**Table S1**. Univariate and multivariate tests for the analysis of temporal changes in density of juvenile rock scallops found within monitored reserve and fishing areas.

| **Tests** | **Value** | **F** | **Num DF** | **Den DF** | **P** |
| --- | --- | --- | --- | --- | --- |
| **Time** |  |  |  |  |  |
| Univariate unadjusted Epsilon | 1 | 6.85 | 4 | 171 | <.0001 |
| Univariate G-G Epsilon | 0.80 | 6.85 | 3.20 | 140.98 | 0.0002 |
| Univariate H-F Epsilon | 0.93 | 6.85 | 3.72 | 163.71 | <.0001 |
| Multivariate Pillai’s Trace | 0.59 | 6.07 | 4 | 41 | 0.0006 |
| **Time X Site** |  |  |  |  |  |
| Univariate unadjusted Epsilon | 1 | 6.13 | 4 | 171 | 0.0001 |
| Univariate G-G Epsilon | 0.80 | 6.13 | 3.20 | 140.98 | 0.0004 |
| Univariate H-F Epsilon | 0.93 | 6.13 | 3.72 | 163.71 | 0.0002 |
| Multivariate Pillai’s Trace | 0.69 | 7.09 | 4 | 41 | 0.0002 |
| **Time X Protection** |  |  |  |  |  |
| Univariate unadjusted Epsilon | 1 | 2.35 | 4 | 171 | 0.05 |
| Univariate G-G Epsilon | 0.80 | 2.35 | 3.20 | 140.98 | 0.07 |
| Univariate H-F Epsilon | 0.93 | 2.35 | 3.72 | 163.71 | 0.06 |
| Multivariate Pillai’s Trace | 0.26 | 2.67 | 4 | 41 | 0.04 |
| **Time X Site X Protection** |  |  |  |  |  |
| Univariate unadjusted Epsilon | 1 | 2.99 | 4 | 171 | 0.02 |
| Univariate G-G Epsilon | 0.80 | 2.99 | 3.20 | 140.98 | 0.02 |
| Univariate H-F Epsilon | 0.93 | 2.99 | 3.72 | 163.71 | 0.02 |
| Multivariate Pillai’s Trace | 0.24 | 2.53 | 4 | 41 | 0.05 |
