# Supplementary material for: Rapid Effects of Marine Reserves via Larval Dispersal
Source: PLoS One. 2009 Jan 8;4(1):e4140. doi: 10.1371/journal.pone.0004140 (PMC2612740; doi:10.1371/journal.pone.0004140)
Supplement: Table S2 — Univariate and multivariate tests for the analysis of temporal changes in density of juvenile black murex found within monitored reserve and fishing areas. (0.05 MB DOC) [file pone.0004140.s002.doc]

**Table S2**. Univariate and multivariate tests for the analysis of temporal changes in density of juvenile black murex found within monitored reserve and fishing areas.

| **Tests** | **Value** | **F** | **Num DF** | **Den DF** | **P** |
| --- | --- | --- | --- | --- | --- |
| **Time** |  |  |  |  |  |
| Univariate unadjusted Epsilon | 1 | 2.03 | 4 | 176 | 0.09 |
| Univariate G-G Epsilon | 0.86 | 2.03 | 4 | 151.89 | 0.10 |
| Univariate H-F Epsilon | 1 | 2.03 | 3.45 | 176 | 0.09 |
| Multivariate Pillai’s Trace | 0.18 | 1.91 | 4 | 41 | 0.12 |
| **Time X Site** |  |  |  |  |  |
| Univariate unadjusted Epsilon | 1 | 3.42 | 4 | 176 | 0.01 |
| Univariate G-G Epsilon | 0.86 | 3.42 | 4 | 151.89 | 0.01 |
| Univariate H-F Epsilon | 1 | 3.42 | 3.45 | 176 | 0.01 |
| Multivariate Pillai’s Trace | 0.28 | 2.95 | 4 | 41 | 0.03 |
| **Time X Protection** |  |  |  |  |  |
| Univariate unadjusted Epsilon | 1 | 2.79 | 4 | 176 | 0.02 |
| Univariate G-G Epsilon | 0.86 | 2.79 | 4 | 151.89 | 0.03 |
| Univariate H-F Epsilon | 1 | 2.79 | 3.45 | 176 | 0.02 |
| Multivariate Pillai’s Trace | 0.32 | 3.28 | 4 | 41 | 0.02 |
| **Time X Site X Protection** |  |  |  |  |  |
| Univariate unadjusted Epsilon | 1 | 3.34 | 4 | 176 | 0.01 |
| Univariate G-G Epsilon | 0.86 | 3.34 | 4 | 151.89 | 0.01 |
| Univariate H-F Epsilon | 1 | 3.34 | 3.45 | 176 | 0.01 |
| Multivariate Pillai’s Trace | 0.29 | 3.02 | 4 | 41 | 0.02 |
