# Supplementary figures and images for: Rapid Effects of Marine Reserves via Larval Dispersal
Source: PLoS One. 2009 Jan 8;4(1):e4140. doi: 10.1371/journal.pone.0004140 (PMC2612740; doi:10.1371/journal.pone.0004140)

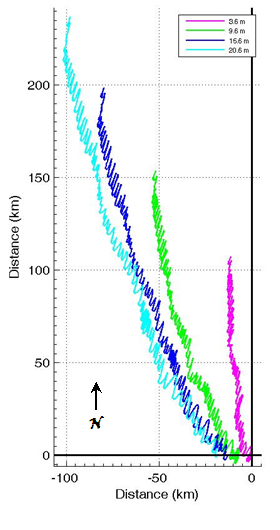

Supplement: Figure S1 — Progressive vector diagrams (PVD) calculated from the ADCP velocity data at 3.6, 9.6, 15.6 and 20.6 m above the bottom (cells 1, 7, 13 and 18) for the period July 6 to August 18 2006. For clarity, successive diagrams are shifted to the left by 5 km. (0.13 MB TIF) [file pone.0004140.s003.tif]

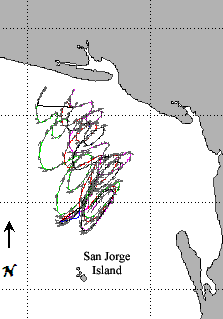

Supplement: Figure S2 — Tracks and velocities of the Microstar surface drifters for the period 19:00 (UT) July 12 to 00:10 July 16 2006. Four of the five drifters shown were redeployed during the period; the exception is the green trace, which is used in Fig. 1 of the article. (0.02 MB TIF) [file pone.0004140.s004.tif]
